# Revisiting the density profile of the fuzzy sphere model for microgel colloids

**DOI:** 10.1039/d4sm01045k

**Published:** 2024-10-07

**Authors:** Frank Scheffold

**Affiliations:** a Department of Physics, University of Fribourg Chemin du Musée 3 1700 Fribourg Switzerland Frank.Scheffold@unifr.ch

## Abstract

Common neutral polymer microgels exhibit an inhomogeneous density profile with a gradual decay that is commonly described using the fuzzy sphere model. The model is based on the idea of convolving the collapsed solid sphere profile with a Gaussian to describe inhomogeneous swelling of the microgel in a good solvent. Here we show that the corresponding density profile in real space used in several recent works – such as in super-resolution microscopy – is different from the fuzzy sphere model, and we explain how to correctly transition between reciprocal space modelling to real space. Our work aims to clarify the application of the model so that errors can be avoided in the future. Our discussion is also crucial when comparing alternative real-space models for the density profile with the established fuzzy sphere model.

## Abstract 2

Microgel density profiles evaluated with the complementary error function and the popular fuzzy sphere model are not compatible with each other.

## Graphical abstract

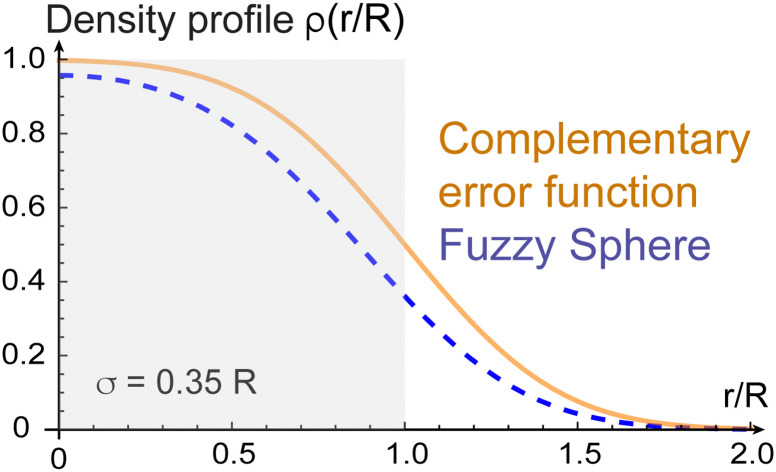

Polymer microgels, also called hydrogels or nanogels, are cross-linked polymer gels where the polymer chain and cross-linking reaction are deliberately stopped to obtain small polymeric beads that can be highly swollen in a solvent medium.^1–6^ Microgels are among the most studied colloidal systems. Some microgels respond to external stimuli, allowing their size and porosity to automatically adapt to environmental changes, which has led to these systems being referred to as ‘intelligent’ or ‘smart’.^7^ Stimuli-responsive behavior can be observed when the polymer's solubility reacts to temperature, pH, ionic strength, or other external triggers. If the solvent quality changes from good to bad, a volume phase transition (VPT) occurs, where the microgel shifts from a highly swollen to a collapsed state.

Among the many microgels that have been studied, those made from thermoresponsive poly(*N*-isopropylacrylamide), or pNIPAM, are probably the most well-known example. The homopolymer poly(*N*-isopropylacrylamide), when dissolved in water, exhibits a lower critical solution temperature (LCST) at around 33 °C.^8^ Above this temperature, the solvent changes from good to bad, causing the polymer to undergo a coil-to-globule transition. For submicron-sized pNIPAM microgels, this transition leads to a rapid shrinking of the colloids by a factor of two or three in size.^9^ While far below the LCST the microgel is composed of more than ninety percent water, after collapse, almost all of the water is expelled.^10^

An important characteristic of pNIPAM microgels is that they acquire a radially inhomogeneous density profile once they are swollen. Instead of a sharp cutoff at the outer perimeter, it has long been known that the density decays more gradually at the interface.^1^ This is generally attributed to the fact that during the synthesis, the cross-linker is consumed more rapidly compared to the NIPAM monomer.^11^ However, even if this were not the case, there must be an interfacial layer, comparable in thickness to the mesh size of the gel,^6,12^ where the polymer chains are dangling and forming brush-like structures, leading to a gradually decaying density profile on several nanometer length scales.^13^ Since many properties of microgels and microgel suspensions are controlled by their interfacial characteristics, it is of prime importance to understand the microgel structure and morphology, particularly at the interface.^14^ In the present work, we will discuss the widely employed fuzzy-sphere model for the microgel density profile and its representations in both real space and reciprocal space. Notably, we will demonstrate that a description of the real space density profile used in several recent papers,^15–19^ including our own,^15^ is not identical to the fuzzy sphere density profile. This work aims to clarify the application of the model so that such inconsistencies can be avoided in the future. This discussion is also important if alternative real-space models for the density profile are proposed^16,20,21^ and compared to the established fuzzy-sphere model.

Early pioneering work by Stieger and co-workers introduced the fuzzy-sphere model for pNIPAM microgels^10^ based on a similar approach by Svaneborg and Pedersen to model the scattering of block copolymer micelles.^22^ The idea is to model the microgels density profile by convolving the profile of a solid sphere with radius *R* with a Gaussian function, where *σ* is the ‘fuzzyness’ parameter given by the standard deviation of the Gaussian.^10,22,23^ They obtain an expression for the microgel scattering form factor:1
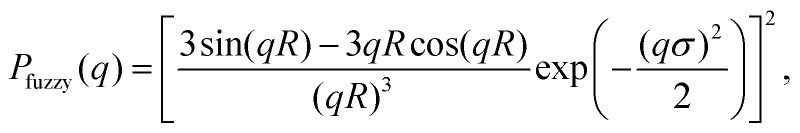
where *P*(*q*) is the square of the normalized scattering amplitude *P*(*q*) = |*A*(*q*)|^2^ with *A*(0) ≡ 1. In three dimensions, the convolution of a solid sphere with a Gaussian function is expressed as follows:2
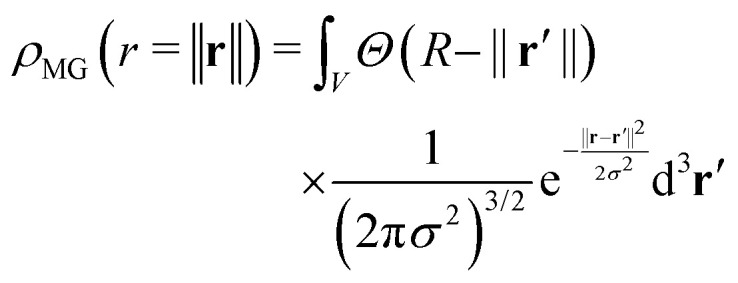
where **r** = (*x*, *y*, *z*) is the position vector, *Θ*(*R* − ‖**r**′‖) is the Heaviside step function, which is 1 inside the sphere (*i.e.*, ‖**r**′‖ ≤ *R*) and 0 outside. In this representation, the normalized microgel density, *ρ*_MG_(*r*), equals 1 for *r* ≤ *R* when *σ* = 0.

The normalized scattering amplitude can be expressed by the microgel radial density profile as follows:3
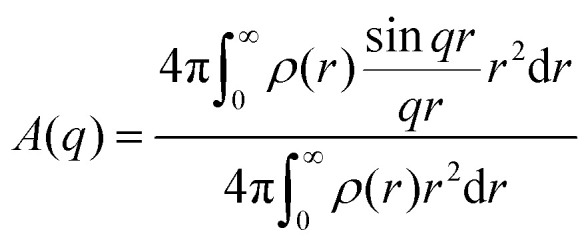
To calculate the actual scattering amplitude, we must multiply by the microgel's polymer mass and the scattering contrast of the polymer in solution, which, for light, is given by the refractive index contrast. The form factor can be averaged over a certain number distribution of particle core radii *R* (and *σ*) to account for the size polydispersity of the particles. For further details, we refer to the literature and textbooks on scattering methods.^10,24^

The fuzzy-sphere model has been applied in numerous studies over the last 20 years in order to establish swelling curves, characterize microgel core–shell architecture, and relate quantities to microgel softness, the rheology of microgel suspensions^25–27^ or their optical properties.^28^ Ratios of the fuzzy shell to the core radius, *σ*/*R*, well above 20% have been reported for highly swollen microgels.^10^ Until not long ago, scattering techniques were the only methods that allowed the nanoscale characterization of microgels in solution, particularly for monitoring the volume phase transition.^29,30^ Direct and indirect inversion methods for scattering has been discussed in the literature, however these inversion algorithms are either ill-posed,^31,32^ and thus very sensitive to experimental noise, or they provide only the pair distance distribution function, which is a measure of spatial density–density correlations.^33^ With the advent of powerful numerical modeling^32,34,35^ and modern fluorescent super-resolution microscopy,^15,36,37^ real-space access to the internal structure and density profile of microgels in solution has become possible,^15^ even allowing for the distinction of different constituents of the microgel at the nanoscale.^36^ These new capabilities have also necessitated the direct modeling of the radially averaged internal density profile of individual microgel beads or density profiles derived from particle ensembles.

Since the fuzzy sphere model proposes to convolve the profile of a solid sphere with a Gaussian, several studies have suggested using the complementary error function as a basis for modeling the radial density profile:^15–19^4
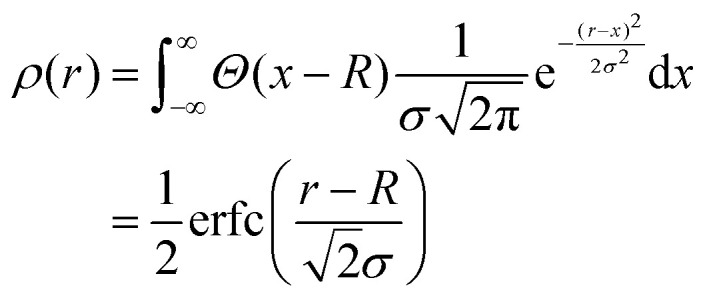
The complementary error function is the result of convolving a step profile with a Gaussian. Since microgels are assumed to be approximately isotropic, extending the convolution from three to one dimension might seem straightforward. However, a closer analysis of eqn (2) shows that, in three dimensions, the convolution does not directly result in a complementary error function.

This discrepancy becomes evident when integrating the density profile. For constant *R* the integral 
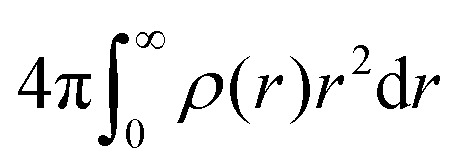
 should be independent of *σ*, but this is not the case when using eqn (4). As *σ* increases, mass is redistributed from the inside to the outside of the sphere perimeter *R* and the redistributed mass *ρ*(*r*) is weighted by *r*^2^. Consequently, the total mass would ‘magically’ increase as the microgel's interface becomes fuzzier. Moreover, in eqn (1), the position of the first minimum remains unchanged as long as *R* is the same, while the minimum shifts to smaller values when using the complementary error function, eqn (4), with eqn (3).

To illustrate the discrepancy between the correct and incorrect model assumptions for fuzzy-spheres in real space, we plot in Fig. 1 different density profiles *ρ*(*σ*/*R*) and form factors *P*(*qR*) for increasing values of *σ*/*R*. In addition, we plot the dependence of the apparent mass of the microgels as a function of *σ*/*R* in Fig. 2. For small values of *σ*/*R* the differences are minor, but increase when *σ*/*R* exceeds 10–15%. In the superresolution work of Conley *et al.*, the largest *σ*/*R* value reported was 13%, meaning the impact of using the complementary error function did not substantially affect the comparison with light scattering data^15^ since the first minimum of *P*(*qR*) would be shifted from *qR* = 4.49 (hard spheres) to *qR* = 4.42 only. However, other scattering studies have reported *σ*/*R* values of 25% and more for more weakly cross-linked microgels,^10^ where the effect would be significant. From Fig. 1(e), we can see that for *σ*/*R* = 0.25, the first minimum is shifted to *qR* = 4.25, corresponding to a more than 5% difference. This value has to be compared to the typical swelling of the core by 20–30%.^10,30^

**Fig. 1:**
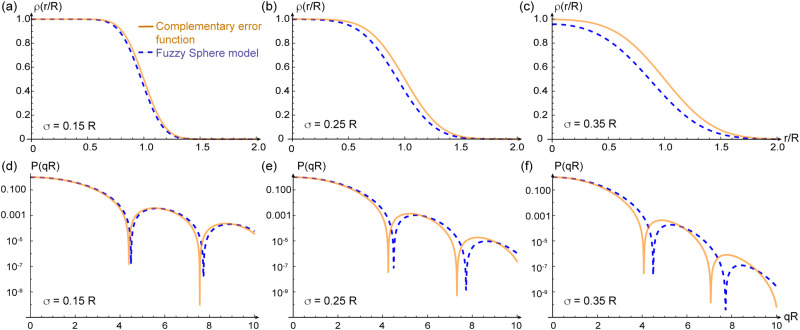
Density profiles *ρ*(*r*/*R*) and scattering form factors *P*(*qR*) for three different values of the fuzzyness parameter *σ*/*R* increasing from left to right. Upper panels (a)–(c) show the radial density profiles and the lower panels (d)–(f) the scattering curves. The solid lines shows the expression evaluated with the complementary error function, while the dashed lines shows the fuzzy sphere predictions.

**Fig. 2:**
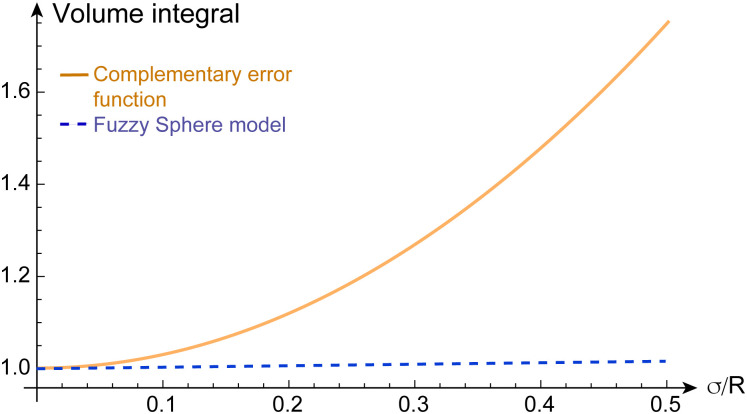
The volume integral 
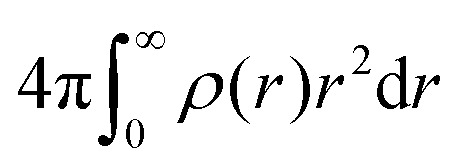
 of the radial density profile, corresponding to the microgel's mass, divided by (4π*R*^3^)/3, as a function of *σ*/*R*. Dashed line: for the fuzzy sphere model, the integral is one in all cases. Solid line: for the case of the complementary error function, the apparent mass increases with *σ*/*R*. For the cases shown in Fig. 1, we find values of 1.0675 for *σ*/*R* = 0.15, 1.1875 for *σ*/*R* = 0.25, and 1.36751 for *σ*/*R* = 0.35.

It is important to emphasize that we make no claim here as to which model, eqn (2) or (4), better describes the real-space properties of the microgels. The complementary error function can certainly be used for this effect if the amplitude of *ρ*(*r*) or the core radius *R* is adjusted such that polymer mass is conserved upon swelling. However, it is important to note that the values of *R* and *σ* obtained from a fit with the complementary error function cannot (!) be compared one-to-one with the values obtained from scattering and the fuzzy sphere model.

In summary, we find that although the fuzzy sphere model provides a convenient and simple analytical description of scattering data, the model does not provide such a simple result in real space. Similarly, although the complementary error function can be easily fitted to experimental or numerical real-space data, there is no corresponding simple solution in reciprocal space. For comparison with scattering data, the density profile predicted by a theoretical model, or determined by a real-space characterization method or simulations, must therefore, in most cases, be numerically integrated using eqn (3).

## Data availability

The data sets shown in Fig. 1 and 2 have been uploaded to the Zenodo repository and are available at https://doi.org/10.5281/zenodo.13895420.

## Conflicts of interest

There are no conflicts to declare.

## References

[cit1] Saunders B. R., Vincent B. (1999). Adv. Colloid Interface Sci..

[cit2] Fernandez-Nieves A. , Wyss H., Mattsson J. and Weitz D. A., Microgel suspensions: fundamentals and applications, John Wiley & Sons, 2011

[cit3] Jiang Y., Chen J., Deng C., Suuronen E. J., Zhong Z. (2014). Biomaterials.

[cit4] Karg M., Pich A., Hellweg T., Hoare T., Lyon L. A., Crassous J., Suzuki D., Gumerov R. A., Schneider S., Potemkin I. I. (2019). et al.. Langmuir.

[cit5] Scheffold F. (2020). Nat. Commun..

[cit6] Plamper F. A., Richtering W. (2017). Acc. Chem. Res..

[cit7] Karg M., Hellweg T. (2009). Curr. Opin. Colloid Interface Sci..

[cit8] Wu C., Wang X. (1998). Phys. Rev. Lett..

[cit9] Scheffold F., Daz-Leyva P., Reufer M., Ben Braham N., Lynch I., Harden J. L. (2010). Phys. Rev. Lett..

[cit10] Stieger M., Richtering W., Pedersen J. S., Lindner P. (2004). J. Chem. Phys..

[cit11] Wu X., Pelton R., Hamielec A., Woods D., McPhee W. (1994). Colloid Polym. Sci..

[cit12] Fernández-Barbero A., Fernández-Nieves A., Grillo I., López-Cabarcos E. (2002). Phys. Rev. E: Stat., Nonlinear, Soft Matter Phys..

[cit13] Meyer S., Richtering W. (2005). Macromolecules.

[cit14] Bergman M. J., Gnan N., Obiols-Rabasa M., Meijer J.-M., Rovigatti L., Zaccarelli E., Schurtenberger P. (2018). Nat. Commun..

[cit15] Conley G. M., Nöjd S., Braibanti M., Schurtenberger P., Scheffold F. (2016). Colloids Surf., A.

[cit16] Bergmann S., Wrede O., Huser T., Hellweg T. (2018). Phys. Chem. Chem. Phys..

[cit17] Ledesma-Motolina M., Braibanti M., Rojas-Ochoa L. F., Haro-Pérez C. (2015). Colloids Surf., A.

[cit18] Ninarello A., Crassous J. J., Paloli D., Camerin F., Gnan N., Rovigatti L., Schurtenberger P., Zaccarelli E. (2019). Macromolecules.

[cit19] Balderas-Cabrera C., Castillo R. (2024). J. Chem. Phys..

[cit20] Mason T., Lin M. (2005). Phys. Rev. E: Stat., Nonlinear, Soft Matter Phys..

[cit21] Boon N., Schurtenberger P. (2017). Phys. Chem. Chem. Phys..

[cit22] Svaneborg C., Pedersen J. S. (2001). Phys. Rev. E: Stat., Nonlinear, Soft Matter Phys..

[cit23] Berndt I., Pedersen J. S., Richtering W. (2005). J. Am. Chem. Soc..

[cit24] Neutrons, X-rays, and Light: Scattering Methods Applied to Soft Condensed Matter, ed. P. Lindner and T. Zemb, Elsevier Science, Amsterdam, 2002

[cit25] Conley G. M., Zhang C., Aebischer P., Harden J. L., Scheffold F. (2019). Nat. Commun..

[cit26] Seth J. R., Cloitre M., Bonnecaze R. T. (2006). J. Rheol..

[cit27] Le Grand A., Petekidis G. (2008). Rheol. Acta.

[cit28] Otten M., Hildebrandt M., Pfeffing B., Voigt V. C., Scheffold F., Hellweg T., Karg M. (2024). Langmuir.

[cit29] Zhou B., Gasser U., Fernandez-Nieves A. (2023). Phys. Rev. E.

[cit30] Reufer M., Daz-Leyva P., Lynch I., Scheffold F. (2009). Eur. Phys. J. E: Soft Matter Biol. Phys..

[cit31] Virtanen O., Mourran A., Pinard P., Richtering W. (2016). Soft Matter.

[cit32] Hazra N., Ninarello A., Scotti A., Houston J. E., Mota-Santiago P., Zaccarelli E., Crassous J. J. (2023). Macromolecules.

[cit33] Glatter O. (1977). J. Appl. Crystallogr..

[cit34] Gnan N., Rovigatti L., Bergman M., Zaccarelli E. (2017). Macromolecules.

[cit35] Moreno A. J., Verso F. L. (2018). Soft Matter.

[cit36] Gelissen A. P., Oppermann A., Caumanns T., Hebbeker P., Turnhoff S. K., Tiwari R., Eisold S., Simon U., Lu Y., Mayer J. (2016). et al.. Nano Lett..

[cit37] Karanastasis A. A., Zhang Y., Kenath G. S., Lessard M. D., Bewersdorf J., Ullal C. K. (2018). Mater. Horiz..

